# Splenectomy vs. splenopexy and analysis of wandering spleen management across age groups: a case report

**DOI:** 10.1097/RC9.0000000000000302

**Published:** 2026-02-16

**Authors:** Anjila Kunwar, Diwakar Koirala, Subhash Chandra Mandal, Tapeshwor Mandal, Vijay Shrestha, Suresh Prasad Shah

**Affiliations:** Department of Surgery, B.P. Koirala Institute of Health Sciences, Dharan, Nepal

**Keywords:** splenectomy, splenic torsion, splenopexy, splenoptosis, wandering spleen

## Abstract

**Introduction:**

Wandering spleen is a rare clinical entity characterized by abnormal splenic mobility due to ligamentous laxity or absence, with an estimated incidence below 0.2%. This condition poses diagnostic and therapeutic challenges, as it ranges from asymptomatic presentations to acute abdomen secondary to torsion or infarction.

**Case Presentation:**

An 83-year-old woman with chronic obstructive pulmonary disease, hypothyroidism, and coronary artery disease presented with 5 days of progressive abdominal pain, vomiting, and distension. Examination revealed a 10 × 10 cm left upper quadrant mass. Computed tomography confirmed an ectopic spleen causing small bowel obstruction. Exploratory laparotomy with splenectomy was performed, revealing a 10 × 12 cm mobile spleen with intact vasculature. Postoperative recovery was uneventful, highlighting the efficacy of surgical intervention in elderly patients.

**Discussion:**

Wandering spleen’s pathogenesis involves congenital (embryonic ligamentous maldevelopment) or acquired (pregnancy, connective tissue disorders) mechanisms. Complications like torsion (reported in 65% of untreated cases) may lead to infarction, rupture, or pancreatitis. While splenopexy is preferred for splenic preservation, splenectomy remains indicated in older patients or vascular compromise, as in our case. Literature review of 10 cases (ages 3–83 years) demonstrates age-dependent management: pediatric cases favor splenopexy, whereas adults more often undergo splenectomy.

**Conclusion:**

Timely imaging and surgical intervention are critical in wandering spleen management. Individualized approaches-prioritizing splenopexy in young patients and splenectomy in elderly or high-risk cases-optimize outcomes. Standardization of surgical techniques and long-term follow-up data are needed to refine treatment protocols.

## Introduction

Wandering spleen is an uncommon medical disorder characterized by excessive splenic mobility due to the weakening or absence of the supporting splenic ligaments. These ligaments typically anchor the spleen in its anatomical position within the left upper quadrant of the abdomen. In this condition, the spleen remains attached primarily by its vascular pedicle, allowing it to displace from its usual location, hence the alternative designations *floating spleen* or *splenoptosis*^[^1^]^. The incidence of the condition is about less than 0.2%^[^1^]^. A wandering spleen may be identified incidentally during physical examination or abdominal imaging as a palpable mass in the abdomen or pelvis. The most significant complication associated with this condition is splenic torsion, which can present in acute, chronic, or intermittent forms due to the spleen’s excessive mobility. Torsion of the splenic vasculature, involving both arterial and venous compromise, leads to splenic enlargement and subsequent ischemic necrosis of the parenchyma^[^2^]^.Splenectomy is the established therapeutic intervention for wandering spleen complicated by hypersplenism. This rare condition may originate from either congenital or acquired pathological mechanisms^[^3^]^.The clinical presentation of wandering spleen varies from acute abdominal emergencies to chronic manifestations including persistent pain and a detectable abdominal mass. Acute symptoms typically arise from splenic torsion, which may extend to involve neighboring visceral structures. Timely recognition and management are crucial for potential splenic preservation. Given the substantial risk of subsequent complications, surgical intervention is invariably indicated, with splenopexy representing the preferred therapeutic approach^[^4^]^.

**HIGHLIGHTS**

- Wandering spleen is a rare cause of abdominal mass and acute abdomen.
- Misdiagnosis with ovarian masses delays timely surgical management.
- Imaging is crucial for accurate diagnosis and treatment planning.
- Splenopexy preserves spleen in young; splenectomy suits elderly cases.
- Early diagnosis and tailored surgery reduce morbidity and complications.

The report has been written in accordance to the SCARE criteria^[^5^]^.

## Case presentation

**Figure 1.**
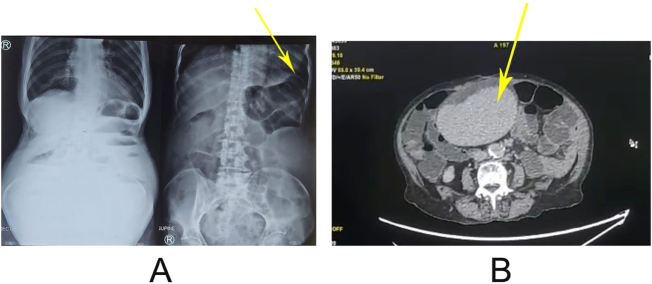
(A) X-rays shows dilated loops and (B) shows ectopic spleen on transverse section.

**Figure 2.**
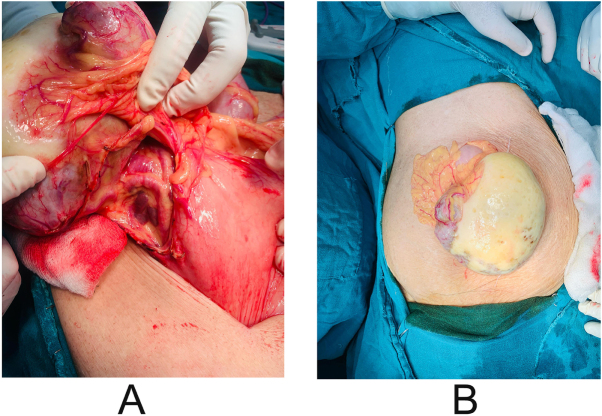
Intraoperative image showing torsion spleen and intact capsule.

**Figure 3.**
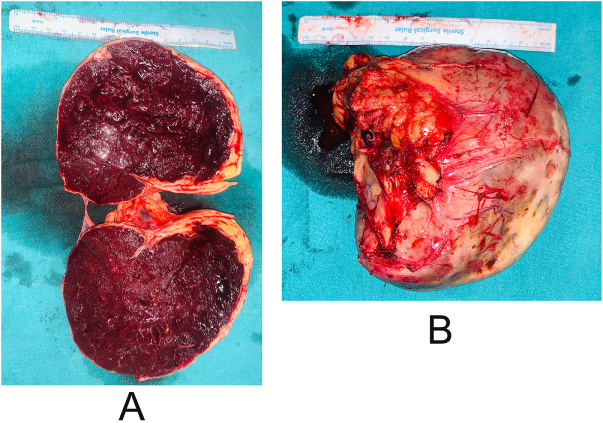
Postoperative image showing hemorrhagic changes in (A), and intact capsule in (B).

A 83-year-old woman with complaints of generalized abdominal pain for 5 days which was acute in onset, dull aching, and moderate to severe in intensity, consistent in nature, gradually progressive, and associated with multiple episodes of vomiting occurring after food intake, accompanied by abdominal distension, loss of appetite, and non-bilious, (this non-bilious vomiting was consistent with the early phase of a partial small-bowel obstruction, during which proximal bowel contents accumulate before significant bile reflux occurs. As the obstruction progressed, abdominal distension and reduced bowel sounds became more prominent) non-foul-smelling vomit with no blood, with no history of fever, yellowish discoloration of sclera and skin, dark-colored urine, clay-colored stool, chest pain, shortness of breath, palpitation, or significant weight loss; the patient had a known history of chronic obstructive pulmonary disease (COPD) managed with long-acting beta-agonist and inhalational corticosteroids, and hypothyroidism managed with thyroxine, along with medication for coronary artery disease (spirolactone, furosemide, and doxycline); on examination, the patient was afebrile with a pulse rate of 80 bpm, blood pressure of 130/90 mmHg, and respiratory rate of 20/min, with a distended abdomen showing generalized tenderness, a palpable mass over the left upper quadrant measuring approximately 10 × 10 cm with no guarding or rebound tenderness, a tympanic note on percussion, and decreased bowel sounds on auscultation, while cardiovascular and respiratory examinations were unremarkable. However, contrast-enhanced computed tomography (CT) imaging of the abdomen and pelvis revealed a homogeneously enhancing ovoid structure, likely representing an ectopic spleen that had migrated from its normal anatomical position. In addition to the axial images, the available CT views demonstrated inferior displacement of the spleen from its normal position in the left hypochondrium, with the organ located in the lower abdomen causing anterior compression of adjacent ileal loops. Although multiplanar reformatted sagittal and coronal images were not available in adequate quality for publication, the radiologic pattern was consistent with a wandering spleen. The spleen showed preserved homogeneous enhancement without evidence of the “whirl sign” or compromised hilar perfusion, suggesting the absence of complete vascular torsion at the time of imaging. CT imaging revealed a homogenously enhancing ovoid structure representing a spleen of normal dimensions that had migrated from its usual anatomical position. The apparent prominence was attributable to its ectopic location rather than true splenic enlargement which due to its ectopic location, displaced bowel loops causing compression of adjacent ileal loops, suggesting a wandering spleen with small bowel obstruction as shown in Figure 1, supported by normal serum electrolytes, creatinine (1.71), urea (99.8), and liver function tests;

The final diagnosis was intestinal obstruction secondary to a wandering spleen with coexisting COPD and hypothyroidism with atrial fibrillation, managed operatively with an exploratory laparotomy and splenectomy on revealing a mobile spleen of normal size (approximately 10 × 12 cm, within the upper physiological range) with a firm consistency. Its displaced position and elongated pedicle produced mild venous congestion, giving the appearance of fullness without true pathological splenomegaly, as shown in Figure 2, intact capsule, present vessels, and parenchyma with a smooth surface as shown in Figure 3. Intraoperatively, the ectopic spleen was located in the lower abdomen where it exerted direct mechanical compression on the adjacent ileal loops, producing a transition point consistent with extrinsic obstruction. No evidence of small-bowel volvulus, mesenteric twist, internal herniation, or adhesive bands was noted. The bowel loops proximal to the compressed segment were dilated, while distal loops were collapsed. Following gentle mobilization and subsequent splenectomy, the compressed segment re-expanded, and the obstruction resolved. A complete run-through of the small bowel was performed, confirming the absence of secondary pathology. A formal microscopic histopathology examination was not performed for this case. Gross intraoperative assessment demonstrated a spleen with intact capsule and patchy hemorrhagic discoloration, suggesting early venous congestion. No gross evidence of infarction, necrosis, or cystic lesions was identified. These findings were consistent with partial torsion rather than complete vascular compromise. Postoperatively, the patient was managed in the surgical ward with intravenous antibiotics and fluids, monitored with a daily drain, remained hemodynamically stable, tolerated a soft diet, and passed stool and flatus without abdominal distension or vomiting. Discharge medications included antibiotics, analgesics, stool softners, and oral theophyllines with inhalational long-acting muscarinic antagonist and corticosteroids with advice to follow a liquid to soft diet; drink plenty of water for 7 days; and watch for abdominal distension, vomiting, or abdominal pain. This case highlights the importance of imaging in diagnosis and the efficacy of surgical intervention, with regular follow-up recommended in surgery, pulmonology, cardiology, and medicine outpatient departments, underscoring the successful management of this rare condition through splenectomy with significant improvement postoperatively.

## Discussion

The initial documented case of wandering spleen dates back to Von Horne’s 1667 report. To date, fewer than 600 instances have been recorded in global medical literature^[^6^]^.The spleen normally resides in the left hypochondrium, where it is stabilized by multiple ligamentous attachments. These include the gastrosplenic, splenorenal, pancreaticosplenic, splenocolic, and splenophrenic ligaments, along with presplenic folds, which collectively anchor the organ to the stomach, kidney, pancreas, colon, and left hemidiaphragm.The pathogenesis of wandering spleen may involve either congenital or acquired mechanisms. Congenital cases result from incomplete fusion of the embryonic septum transversum with the posterior abdominal wall, leading to underdeveloped or lax supporting ligaments^[^7^]^. Acquired forms develop secondary to conditions causing ligamentous laxity, including pregnancy or connective tissue disorders. While the displaced spleen may be located in any abdominal or pelvic quadrant, it most frequently remains in left-sided regions, suspended solely by an elongated vascular pedicle^[^7^]^.In cases of ligamentous insufficiency, the spleen may become displaced from its anatomical position to various locations within the abdominal or pelvic cavities. This pathological mobility can result in elongation and subsequent torsion of the splenic vascular pedicle, comprising the splenic artery and its six venous tributaries. Vascular compromise from such torsion may induce splenic congestion, ultimately leading to parenchymal engorgement and splenomegaly^[^8^]^.Progressive splenomegaly may exacerbate splenic hypermobility, increasing the risk of torsion a critical precipitating factor for splenic infarction. The combination of an elongated vascular pedicle and excessive splenic mobility predisposes to hilar-axis torsion, representing a potentially fatal complication of wandering spleen. The clinical consequences vary according to the extent of torsion, ranging from localized splenic infarction to severe sequelae including gangrenous transformation, abscess development, peritonitis, bowel obstruction, or splenic rupture^[^9^]^.Torsion involving both the splenic vascular pedicle and the pancreatic tail at the splenic hilum may result in dual pathology: pancreatitis combined with ischemic compromise of the distal pancreas. In the present case, the mechanism of intestinal obstruction was purely compressive rather than torsional. The inferiorly displaced spleen created localized mass effect over the ileal loops, producing a mechanical transition point without rotation of the mesentery. This reinforces that wandering spleen may precipitate obstruction either via mass effect, as observed in our patient, or less commonly through volvulus of adjacent bowel segments. In the present case, only gross examination was available, which revealed patchy hemorrhagic areas consistent with early vascular congestion. The absence of frank necrosis or devitalized splenic tissue on visual inspection supported the diagnosis of partial torsion. Although formal histopathology was not performed, the operative findings aligned with early compromise rather than complete strangulation. The clinical spectrum of wandering spleen ranges from incidental asymptomatic detection to acute abdominal emergencies. In adult populations, the majority of cases remain clinically silent, with diagnosis typically occurring either during routine physical examination through identification of a mobile, firm abdominal mass demonstrating pathognomonic notched borders, or as an incidental finding on imaging studies performed for unrelated indications^[^10^]^. In our patient, the spleen was not enlarged beyond normal adult limits. The perceived increase in size resulted from its ectopic location combined with early venous congestion secondary to partial pedicle torsion. Differentiating true splenomegaly from positional fullness is essential to avoid overestimating disease severity. Although small-bowel obstruction typically produces bilious vomiting, early or partial obstruction may initially present with non-bilious emesis, as occurred in our patient. This reflects impaired gastric and duodenal emptying before proximal migration of bile, a pattern described in evolving mechanical obstruction. Surgical intervention represents the definitive management for wandering spleen, as conservative approaches demonstrate a 65% complication rate. While splenectomy^[^11^]^ was historically the standard treatment irrespective of torsion status, contemporary practice favors splenic preservation through splenopexy^[^12^]^. This paradigm shift reflects both improved recognition of the spleen’s immunological functions and concerns regarding post-splenectomy sepsis, particularly in pediatric patients. The medical literature describes multiple splenopexy techniques, including

1. Diaphragmatic fixation via suturing^[^13^]^
2. Retroperitoneal anchorage to the posterior abdominal wall^[^14^]^
3. Omentoplasty-assisted stabilization (with or without synthetic mesh reinforcement using materials such as Dacron or Prolene)^[^15^]^

In our case, however, after careful consideration of the patient’s age-related risk factors, the surgical team opted for splenectomy rather than splenic preservation. In pediatric and younger patients, splenic preservation remains the cornerstone of management, as their immunologic risk after splenectomy is significantly higher. Splenopexy performed either through open or increasingly through laparoscopic techniques is therefore recommended when the spleen is viable and the procedure is undertaken electively. Laparoscopy offers the advantages of reduced postoperative pain and faster recovery. Additionally, children with wandering spleen may have associated conditions such as connective-tissue disorders or congenital ligamentous abnormalities, which further support the preferential use of splenopexy in this group. In contrast, in elderly patients such as ours, splenectomy remains appropriate due to increased operative risk, higher likelihood of vascular compromise, and lower long-term immunologic vulnerability.

Below are few cases of wandering spleen with their treatment and prognosis for better comparison with the literature as shown in Table 1.

**Table 1:** Shows comparison to different cases with their prognosis

S. No.	Authors	History of patient	Treatment	Prognosis
1	Hashiguchi *et al*^[^2^]^	A 35-year-old woman with no any abdominal symptoms, WS diagnosed 20 years back suddenly developed pain 2 months after delivery	Splenectomy	No complications post surgery, weight of the spleen 350 g, Good prognosis
2	Koliakos *et al*^[^3^]^	A 25-year-old woman with recurrent abdominal pain with raised serum lipase and CRP	Exploratory laparotomy with splenectomy	No complications post surgery, Discharged 7 days after surgery
3	Lebeul *et al*^[^16^]^	A 34-year-old woman with acute abdominal pain in the left flank region	Laparoscopic Splenopexy	Good prognosis
4	Midha *et al*^[^17^]^	A 45-year-old woman with left abdominal pain, dull aching for 2 months	Exploratory laparotomy with splenectomy	500 g Spleen, Uneventful recovery and discharged on 5th post-op day. Complicated by splanchnic thrombosis on 12th post-op day and discharged after 6 days of ward following relook Laparotomy and resection of ischemic intestine admission and reversal of stoma after 68 days
5	Richman *et al*^[^18^]^	A 38-year-old woman with right abdominal pain for 2 days	Laparoscopic splenectomy	Uneventful recovery
6	Shibiru *et al*^[^19^]^	A 39-year-old woman with lower abdominal pain for 1 year	Emergency laparotomy followed by splenectomy	Uneventful recovery, discharged on 3rd post-op day with pentavalent vaccination in the 3rd week
7	Steinberg *et al*^[^12^]^	Case 1: A 4-year-old boy with abdominal pain, vomiting and fever	Case 1: Splenopexy to the posterior abdominal wall	Case 1: Discharged on 7th post-op day
				Case 2: Discharged on 7th post-op day
				Case 3: Discharged on 7th post-op day
				Case 4: Discharged on 4th Post op day
			Case 2: Laparotomy with splenopexy with Ti-Cron suture	Case 5: Discharged on 5th post-op day
		Case 2: A 6-year-old boy with abdominal pain for 1.5 months		
		Case 3: A 12-year-old boy with 4 months history of intermittent abdominal pain		
		Case 4: A 15-year-old girl with 10 months history of abdominal pain and bilious vomiting		
		Case 5: A 4.5-year-old girl with epigastric pain and vomiting		
			Case 3: Laparotomy followed by omentopexy	
			Case 4: Exploratory laparotomy followed by splenectomy(due to absent blood flow to spleen despite detorsion on Doppler)	
			Case 5: Treatment for pancreatitis followed by Extraperitonealsplenopexy	
8	Wang *et al*^[^10^]^	A 3-year-old boy with 2 days history of abdominal pain and vomiting	Emergency laparotomy with splenectomy	Discharged on 7th post-op day.
9	Wester *et al*^[^20^]^	A 36-year-old woman with 1 day history of abdominal pain and vomiting	Conservative management due to evaluation for liver transplantation	Uneventful
10	Moore *et al*^[^21^]^	A 21-year-old woman with epigastric pain and vomiting	Exploratory laparotomy with gastric decompression	Patient discharged on 10th post-op day.

CRP, C-reactive protein; WS, wandering spleen.

The analysis of these cases reveals important trends in wandering spleen management. Pediatric patients tend to undergo spleen-preserving techniques, while adults more frequently receive splenectomies, reflecting differing risk profiles. Acute presentations typically require urgent intervention, whereas chronic cases allow for planned procedures. Surgical approaches vary from simple suturing to more complex mesh reinforcements, though standardized techniques have yet to be established. Complications such as vascular thrombosis and post-splenectomy infections underscore the need for careful patient selection. The decision between splenopexy and splenectomy ultimately depends on multiple factors including patient age, symptom severity, and splenic viability, with preservation preferred when anatomically feasible.

## Conclusion

Wandering spleen, though rare, remains a clinically significant entity requiring timely recognition and individualized management. The present case of an 83-year-old woman misdiagnosed with an ovarian mass underscores the diagnostic challenges and the importance of including ectopic spleen in the differential diagnosis of abdominal masses, particularly in female patients. Imaging plays a central role in establishing an accurate diagnosis and guiding management. While splenopexy is preferred for younger patients to preserve splenic function, splenectomy remains appropriate in elderly individuals or those with compromised vascular supply, as demonstrated in this case. The wide spectrum of presentations – from incidental findings to acute emergencies – demands tailored treatment strategies and vigilant postoperative care, given the risks of torsion, infarction, and post-splenectomy sepsis. The absence of standardized splenopexy techniques highlights the need for further research to optimize surgical outcomes. Ultimately, early diagnosis, appropriate intervention, and multidisciplinary follow-up are essential to improving prognosis. This case reinforces that wandering spleen, despite its rarity, should be carefully considered in the evaluation of unexplained abdominal pain or mass lesions, particularly when supported by imaging evidence.

## Data Availability

All data relevant to the case are included in the article. No additional data are available.

## Research registration unique identifying number (UIN)

Not applicable for case reports.
